# Spectroscopic and calorimetric study of the interaction between Nile blue and double-stranded RNA

**DOI:** 10.1016/j.bbrep.2024.101899

**Published:** 2024-12-14

**Authors:** Md Dulal Hossain Khan, Ramya Ayyalasomayajula, Mare Cudic, Renjie Wang

**Affiliations:** Department of Chemistry and Biochemistry, Florida Atlantic University, 777 Glades Road, Boca Raton, FL, 33431, USA

**Keywords:** Nile blue, poly(A·U), poly(I·C), Interaction property investigation

## Abstract

Nile blue has been widely used in histological staining, fluorescence labeling, and DNA probing, with its intercalation behavior into the DNA helix being well documented. Here, we present a comprehensive investigation to address a current knowledge gap regarding the binding properties of Nile blue to two types of double-stranded RNA (dsRNA): poly(A·U) and poly(I·C), using various biophysical techniques. Absorption and fluorescence spectroscopic studies suggest a significant binding interaction between Nile blue and the two designated dsRNAs, specifically indicating an intercalation binding mode with poly(A·U) and demonstrating a noticeably higher binding affinity compared to poly(I·C). The binding stoichiometry was further determined by Job's plot to be 0.47 for poly(A·U) and 1.0 for poly(I·C). The increased relative viscosity and changes in the circular dichroism (CD) ellipticity of dsRNA after interacting with Nile blue indicate the stacking of Nile blue dyes between the RNA duplexes. These changes suggest a conformational alteration of the dsRNAs and confirm the intercalation mode of binding. The thermal dynamic analysis demonstrates that both binding were favored by negative enthalpy and primarily driven by the hydrophobic effect.

## Highlights

• Interaction properties between Nile blue and dsRNA was comprehensively studied.
• Nile blue showed higher binding affinity with poly(A·U) than with poly(I·C).
• Both bindings were enthalpy-favored and mainly driven by the hydrophobic effect.
• Advance RNA research and aid in developing therapeutic strategies.

## Graphical abstract

**Figure:**
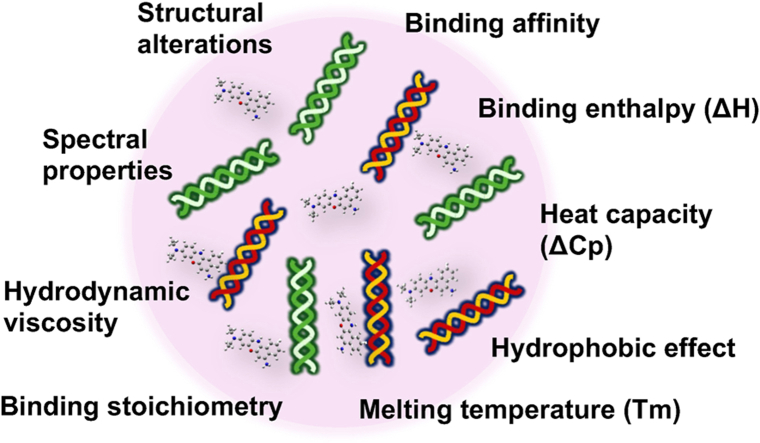

## 1 Introduction

Nile blue is a nontoxic phenoxazine dye with proven versatility in various areas. Its applications span from histological staining of biological samples and serving as a visible indicator in DNA electrophoresis gels to its utilization in diverse optical and electrochemical sensors [[1], [2], [3]]. This multifunctionality is attributed to its long-wavelength absorption, high fluorescence quantum yield, electrochemical activity, and cost-effectiveness [4,5]. Significantly, Nile blue holds approval from the Food and Drug Administration (FDA) for human use [2]. It exhibits selective localization within animal tumors, augmenting its potential as a photosensitized agent for cancer photodynamic therapy [6,7]. Hence, the interaction between Nile blue and DNA has been extensively examined through a variety of methods and perspectives. Generally, there are three types of interactions between organic dyes and nucleic acids: intercalative binding into the base pairs of nucleic acids, groove binding the major or minor groove of nucleic acids, and long-range assembly on the molecular surfaces of nucleic acids. As part of the phenoxazine dye family, Nile blue possesses a planar rigid aromatic system that has been demonstrated to enhance the intercalation of Nile blue into the comparatively non-planar interior of the DNA helix [8]. Consequently, Nile blue has been developed as a versatile DNA probe, facilitating DNA analysis through colorimetric, absorption, and fluorescence methods [[9], [10], [11]]. Meanwhile, certain studies have indicated that the interaction modes between Nile blue and DNA were not uniform. Gattuso and Hirakawa et al. using molecular modeling study coupled with spectroscopy experiments concluded that Nile blue can intercalate into DNA stacked base pairs and also bind into DNA minor groove. Under visible light, Nile blue induces irreversible DNA lesions through photoinduced electron transfer from the dye to a nearby guanine [12,13]. Mukti et al. further demonstrated that Nile blue and DNA interactions were concentration dependent. At low concentrations, Nile blue initially engages cooperatively with the minor groove of DNA, triggering a conformational change in the DNA helix and generating a gap between the base pairs. Nile blue subsequently intercalates into the DNA base pairs as concentrations rise without impacting the molecules bound to the minor groove [14]. Huang et al. have once reported the long-range assembly of Nile blue on the molecular surface of DNA [15]. In contrast to the wealth of information available on the DNA binding of Nile blue, comprehending the interaction between Nile blue and RNA has been overlooked.

While our understanding of RNA's functional behavior remains rudimentary, compelling evidence supports its pivotal role in various cellular processes [16,17]. On one hand, RNA damage plays a significant role in the initiation of various well-known diseases [18], such as Parkinson's, Alzheimer's, and Lewy body dementia; also, RNA serves as the genetic material for viruses, leading to infections such as HIV, Ebola, hemorrhagic fever, influenza, hepatitis C, respiratory diseases, and more [19]. On the other hand, small RNAs, including microRNAs, small interfering RNAs, small nucleolar RNAs, and small nuclear RNAs, exert control over transcription and translation processes and have emerged as valuable tools for targeting RNA as a molecule for therapeutic intervention [20]. Moreover, the successful development and application of the fist mRNA utilized in COVID-19 vaccines have played a pivotal role in alleviating the impact of the COVID-19 pandemic. The advancements in RNA research have substantially progressed, offering significant benefits to our lives [21]. RNAs exhibit a myriad of intricate secondary and tertiary structures, adopting both trans and cis conformations with variations in base-pairing modes, ranging from Watson-Crick to diverse canonical forms [22,23]. Double-stranded RNA (dsRNA) is naturally generated by cells during regular gene expression through intra- and intermolecular RNA interactions, contributing to various biological processes [24].Additionally, dsRNA is a common byproduct in the replication of many viruses replication in mammalian cells [25]. The investigation into the interplay between dsRNA and small molecules has sparked broad research interests, driven by a desire to understand the cellular functional pathways associated with dsRNA and delve into developing RNA-targeted drugs [[26], [27], [28], [29]].

Given the adaptability of Nile blue in DNA research and the importance of advancing dsRNA exploration, we conducted a comprehensive examination of the interaction between Nile blue and different synthetic dsRNA sequences poly(A)·poly(U) and poly(I)·poly(C). Various spectroscopic and thermal dynamic techniques were employed, with a particular focus on characterizing interactional affinity and conformational changes. The results contribute essential biophysical insights into the interaction between Nile blue and dsRNA, holding the potential to advance the field of RNA studies. Furthermore, by combining detailed mechanistic insights with robust experimental approaches, this study not only addresses a significant knowledge gap, the largely overlooked binding interactions of Nile Blue with dsRNA, but also establishes a framework for future investigations into RNA-ligand interactions. This work positions itself as a foundational study that opens new research avenues in nucleic acid biophysics and the development of RNA-based therapeutic materials.

## 2 Experiment section

### 2.1 Materials

Nile blue, poly A.U and poly I.C, sodium cacodylate trihydrate, and agarose gel were purchased from Sigma Aldrich. Na_2_EDTA and NaCl were purchased from Fisher Scientific. A pH 7.0 cacodylate buffer (10 mM sodium cacodylate Na(CH_3_)_2_AsO_2_.3H_2_O, 0.1 mM Na_2_EDTA, and 25 mM NaCl) was employed in this study to prepare dsRNA solutions. Concentration of poly A.U and I.C were checked using molar extinction coefficients values (*ε* in M^−1^ cm^−1^) of 14280 at 260 nm for poly(A).poly(U), 10000 at 260 nm for poly(I).poly(C) [30]. Prior to use, each buffer solution was made using DI water and passed through a 0.45 μm millipore membrane filter.

### 2.2 Absorbance titration and optical thermal melting study

The absorption investigation was conducted using an Agilent Cary 3500 spectrophotometer (USA), which was mounted on a thermoelectric regulating cell holder. The experiments were carried out in quartz cuvettes with a route length of 1 cm. The titration processes were previously explained in great depth [31]. Generally, different concentrations of RNA polynucleotide were combined with a constant dye concentration to produce different [dsRNA]/[dye] ratios. The buffer content of the reference cell was the same as that of the sample cell. The titrations were carried out until the spectrum changes reached saturation.

Absorbance versus temperature profiles (optical melting curves) of the complexes were measured on using the same spectrophotometer and the helix melting of the double helical forms of RNA was observed in the presence and absence of the dye by recording changes in UV absorbance at 260 nm at various temperatures. The rate of heating was 0.5 °C per minute. Melting curves allowed the monitoring of the hyperchromic change and estimation of melting temperature, T_m_, the midpoint of the hyperchromic transition.

### 2.3 Fluorescence spectral and quencing study

Steady state fluorescence spectrum measurements were carried out in fluorescence-free quartz cuvettes with a path length of 1 cm using a PerkinElmer LS55 Fluorescence Spectrometer (UK). For Nile blue, an excitation wavelength of 631 nm was used, and an excitation and emission band pass of 5 nm was maintained while the emission intensity was tracked between 640 and 800 nm. Fluorescence spectra were recorded without correction [32]. The experiment aimed to potassium iodide (KI) was employed as fluorescence quencher to investigate intercalative binding between RNA and dye. KCl were added to maintain a constant ionic strength during the experiment. While tracking the fluorescence intensity as a function of KI, the consistent [dsRNA]/[dye] ratio was maintained. Stern-Volmer plots were used to display the relative fluorescence intensity (F_o_/F) against [KI].

The Stern–Volmer equation is as

$$F_{O}/F=1+K_{SV\;}\left[KI\right]$$

where $F_{o}$ is the fluorescence intensity in the absence of quencher while F is the fluorescence intensity in the presence of quencher, and K_SV_ is Stern–Volmer constant [33,34].

### 2.4 Binding constant and binding stoichiometry analysis

The concentration of dsRNA was adjusted and added to a fixed, concentrated dye solution until saturation was reached in both the fluorescence and absorption spectral titrations. The Benesi–Hildebrand plots were used to determine the equilibrium constants from the spectral data as

$$\frac{1}{\Delta A}=\frac{1}{\Delta A_{\max}}+\frac{1}{K_{BH}\left(\Delta A_{max}\right)}\times \frac{1}{\left[M\right]}$$

where M is the RNA concentration and ΔA is difference between absorbance of dye and RNA-dye complex at $\lambda_{\max}$ and $K_{BH}$ is Benesi–Hildebrand constant [35].

The RNA duplex binding stoichiometry of Nile blue was determined by utilizing Job's approach [36]. The solutions were made at a steady temperature, with the sum of the concentrations of duplex and Nile blue remaining constant at 10 μM despite variations in their individual concentrations. The intensity of fluorescence ($\lambda_{\max}$ = 670 nm) was measured, and the mole fraction of Nile blue was plotted against the relative difference in fluorescence intensity at 670 nm. The mole fraction of Nile blue in complex was obtained from the plot's break point. The stoichiometry was obtained in terms of duplex: NB [(1 − $X_{NB}$)/ $X_{NB}$] where $X_{NB}$ denotes the mole fraction of Nile blue at the plot's break point. The results reported here are averages of at least three measurements.

### 2.5 Viscosity measurement

A semi-micro capillary viscometer of the Cannon-UBBELOHDE-MICRO VISCOMETER from Cannon Instruments Co. (State College, PA, USA) was used to measure the flow timings. It was positioned vertically in a bath of constant temperature (293.15 ± 1) K. Using an electronic stopwatch, the flow timings of dye-RNA complexes were recorded [37]. Relative viscosities $\frac{{\eta ՛}_{sp}}{\eta_{sp}}$ for RNA either in the presence or in the absence of the dye were calculated from the relation [38].

$$\frac{{\eta ՛}_{sp}}{\eta_{sp}}=\frac{\left[\left(t_{complex}-t_{0}\right)/t_{0}\right]}{\left[\left(t_{control}-t_{0}\right)/t_{0}\right]}$$

Where $t_{0}$, $t_{control}$ and $t_{complex}$ are the flow time of buffer, dsRNA and dsRNA-Nile blue complexes respectively.

### 2.6 Circular dichroism spectroscopy

Circular dichroism (CD) measurements were performed using a PC-driven JASCO J-815 spectropolarimeter (Jasco International Co., Japan) equipped with a rectangular quartz cuvette with a 1 cm path length and a Jasco PTC-348WI temperature control unit. CD spectra across the wavelength range of 220–320 nm was recorded at a scan speed of 100 nm/min, with a response time of 1 s and a sensitivity of 100 millidegrees at 20 °C. Each spectrum was averaged from five successive accumulations, baseline-corrected, and smoothed within the allowed limits using the unit's spectrum analysis software. The final CD spectra were expressed in molar ellipticity (deg·cm^2^·dmol⁻^1^).

### 2.7 ITC study

All excess-site isothermal titration calorimetry measurements were performed on a PEAQ-ITC MicroCal (MicroCal) instrument equipped with automated washing module. The sample cell was loaded with 280 μL of 30 μM dsRNA, while the sample syringe contained 120 μM Nile blue solution buffered with 10 mM sodium cacodylate at pH 7.0. The syringe rotation speed was 500 rpm, and the reference power was set to 40 μcal/s with an interval of 160 s between each injection. The first-injection (0.4 μL) was followed by five subsequent injections, each with a volume of 2 μL of Nile blue solution, into the dsRNA in the sample cell at 288, 293, and 298 K, respectively. Each injection generated a heat burst curve (microcalories per second versus time). The heat burst curves from the titration of RNA with Nile blue were integrated by using Origin 7.0 software (MicroCal), and then subtracted point-by-point from the corresponding enthalpy values obtained in the control experiment (Nile blue in buffer). This yielded the corrected enthalpy changes (ΔH) for the RNA-Nile blue interaction.

## 3 Results and discussion

### 3.1 Binding mode and affinity analysis: UV–Vis absorption and fluorescence spectroscopic study

**Fig. 1:**
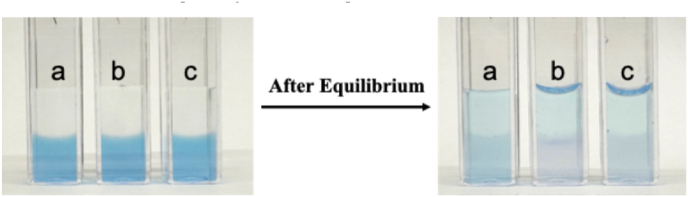
Pictures of Nile blue (10 μM) dissolved in agarose gel (lower phase) interacting and reaching equilibrium with buffer (a), poly(A·U) buffer solution (b), and poly(I·C) buffer solution (c).

The binding between Nile blue and dsRNA was intuitively observed through the competitive retention of Nile blue in both the presence of dsRNA and agarose gel. A 10 μM solution of Nile blue was initially dissolved in agarose gel. The upper layer solutions consisted of buffer, poly(A·U) solution, and poly(I·C) solution, in which Nile blue tended to diffuse into the upper layer buffer until equilibrium. When dsRNA was introduced into the upper-layer buffer solutions, a lower concentration of Nile blue molecules was observed in the agarose gel phase compared to the buffer alone as shown in Fig. 1a. Additionally, the agarose gel phase interacting with the upper buffer solution containing poly(A·U) (Fig. 1b) appeared lighter in color than when interacting with the upper buffer solution containing poly(I·C) (Fig. 1c).

**Fig. 2:**
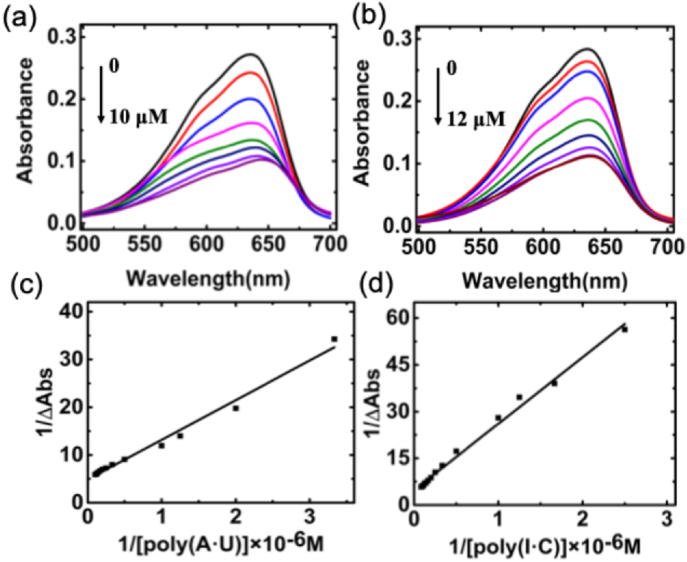
Representative absorption spectral changes of Nile blue (10 mM) treated with 0.5, 0.8, 2, 4, 6, 8,10 μM of poly(A·U) (a) and 0.5, 1, 3, 5, 7, 9, 11, and 12 μM poly (I·C) (b) in 10 mM Cacodylate buffer (pH 7.0).Their corresponding Benesi–Hildebrand plots are shown in (c) and (d).

The binding mode and strength between Nile blue and dsRNA were further assessed by monitoring changes in absorbance intensity and peak shifts during titration with varying dsRNA concentrations. The UV–visible absorption technique has been extensively demonstrated as an effective method for determining the complex formation between small molecules and biomacromolecules. In instances where small molecules interact with double-stranded nucleic acids through intercalation, the π∗ orbital of the intercalated molecule can form a coupling interaction with the π orbital of the base pairs, consequently reducing the π-π∗ transition energy and inducing a bathochromic shift (red shift). Concurrently, the partially filled coupling π orbital contributes to a reduction in transition probabilities, leading to concomitant hypochromism. The upper panel of Fig. 2 presents the representative absorption spectra of Nile blue subsequent to the addition of a series of poly(A·U) and poly(I·C). Notably, both poly(A·U) and poly(I·C) induce significant hypochromism in the absorption spectra of Nile blue, displaying a positive correlation. However, it is noteworthy that the bathochromism resulting from the binding with poly(I·C) is comparatively smaller than that induced by the binding with poly(A·U). Upon reaching saturation binding, the absorption spectra of Nile blue exhibit 63 % hypochromism with a 10 nm red shift when binding with poly(A·U) at a 1:1 ratio. Nile blue demonstrates 63 % hypochromicity with a 5 nm red shift when binding with poly(I·C).

The representative BH plot of each complexation was shown in the below panel of Fig. 2. The binding affinity values of Nile blue to poly(A·U) and poly(I·C) are calculated as 5.86 × 10^5^ M^−1^ and 1.16 × 10^5^ M^−1^, respectively (Table 1). Nile blue exhibits an order of magnitude higher binding affinity to poly(A·U) than poly(I·C). Moreover, an isosbestic point at 675 nm was discerned in the spectral profile during the interaction with the poly(A·U). This observation indicates the presence of spectroscopically distinct chromophores, namely, free and bound species, demonstrating equilibrium conditions in the interaction between the dye and RNA at any wavelength. In contrast, the complexation of Nile blue with poly(I·C) did not manifest any isosbestic point, possibly suggesting a reduced occurrence of a classical intercalation phenomenon. Overall, the results of the absorption spectral study suggest a significant interaction between Nile blue and the dsRNAs, indicating an intercalation binding mode with poly(A·U) and exhibiting a noticeably higher binding affinity compared to poly(I·C).

**Table 1:** Binding constant of Nile blue-dsRNA association from spectroscopic data.

	Spectrophotometry K_BH_ × 10^−5^ (M^−1^)	Spectrofluorimetry K_BH_ × 10^−5^ (M^−1^)
poly(A·U)	5.86	2.11
poly(I·C)	1.16	1.13

**Fig. 3:**
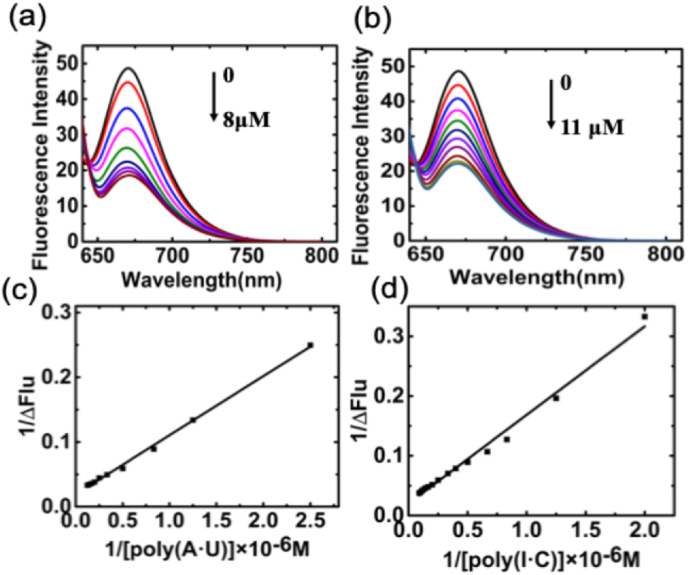
Representative steady state fluorescence emission spectrum changes of Nile blue (10 mM) treated with 0.5, 0.8,1, 2, 3, 4, 6, 8 μM of poly(A·U) (a) and 0.5, 0.8, 1, 2, 3, 4, 5, 7, 9,11 μM poly (I·C) (b) in 10 mM cacodylate buffer (pH 7.0) and their corresponding Benesi–Hildebrand plots (c) and (d).

The fluorescence spectra changes of Nile blue, induced by binding with dsRNA, were monitored during the titration process with increasing concentrations of dsRNA. The results are presented in Fig. 3. Nile blue is a highly fluorescent compound with peak intensity at a wavelength of 670 nm, and the fluorescence undergoes a noticeable decline upon adding dsRNA. This aligns with prior research on the interaction between fluorescence molecules DNA and RNA. While the observed reduction in fluorescence has been ascribed to various factors, including incomplete screening from water molecules [39], improved environmental hydrophobicity [40], or photosensitization [41] via an electron transfer mechanism, the fluorescence decrease signifies the intercalated state of the Nile blue molecule within the dsRNA base pairs [42]. In a 10 μM Nile blue solution, fluorescence reduction reached saturation when the poly(A·U) concentration increased to 8 μM. However, achieving the lowest fluorescence required a concentration of 11 μM poly(I·C). This observation reaffirms the higher binding affinity of Nile blue for poly(A·U) compared to its affinity for poly(I·C). The binding constant K_BH_ for the interaction between Nile blue and the two dsRNAs was further determined using the Benesi-Hildebrand plot based on the fluorescence spectra. The obtained results, as detailed in Table 1, closely aligned with those calculated through absorption spectrophotometric analysis.

### 3.2 Fluorescence quenching based binding mode determination

**Fig. 4:**
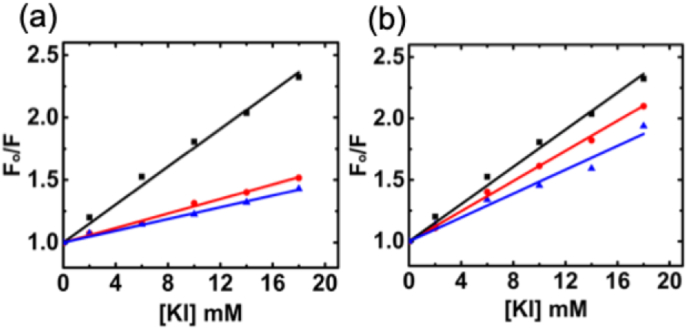
Stern–Volmer plots for the quenching of Nile blue fluorescence by KI in the absence (black) and in presence of poly (A.U) (a) and poly (I.C) (b) in 10 mM cacodylate buffer of pH 7.0 at 25 °C (red curve: 0.5 M ratio of dsRNA-Nile blue complex; blue curve:1.0 M ratio of dsRNA-Nile blue complex).

The strong ion pairing between the positively charged dye and the small anion tends to lead to the formation of dimers or aggregates, as it minimizes the electrostatic repulsion between cationic dyes [43]. Both ion pairing and aggregation are well-established phenomena that induce fluorescence quenching, often attributed to mechanisms such as intermolecular electron transfer [44]. Due to its positive charge, cationic dye is anticipated to bind with the small anionic quencher. In instances of intercalation, the dye molecule positions itself between the base pairs of an RNA duplex, rendering it inaccessible to the quencher. Additionally, RNA's negatively charged phosphate backbone limits the penetration of an anionic quencher into the helix, thereby shielding dye molecule from quenching by the anionic quencher. Therefore, the Stern-Volmer quenching constant (K_SV_) calculated based on fluorescence quenching study provides additional information for the exact location of cationic dye binding to dsRNA. The compound intercalating with RNA exhibits a lower K_SV_ than that of groove binding or free states. As demonstrated in Fig. 4, the quenching effect of iodide ions on free Nile blue was first confirmed, with the K_SV_ calculated as 77 M^−1^. Subsequently, the quenching effect of iodide ions on Nile blue bound to poly(A·U) and poly(I·C) was further examined. The corresponding K_SV_ values were 29 M^−1^ and 23 M^−1^ for 0.5 and 1 M ratio of poly(A·U)-Nile blue complex and 61 M^−1^ and 48 M^−1^ for 0.5 and 1 M ratio of poly(I·C)-Nile blue complex. In both cases, K_SV_ values were decreased from free Nile blue to complex formation with RNA. A lower K_SV_ value, in comparison to free Nile blue, suggests that Nile blue binds to both forms of dsRNA through intercalation. Additionally, Nile blue demonstrates more effective intercalation penetration in poly(A·U) compared to poly(I·C). Therefore, the results uniquely demonstrate intercalation as the dominant binding mode, with poly(A·U) showing significantly higher binding affinity compared to poly(I·C). This underscores the sequence-specific nature of Nile blue's interaction, a characteristic not previously documented for RNA.

### 3.3 Binding stoichiometry analysis via Job's plot

**Fig. 5:**
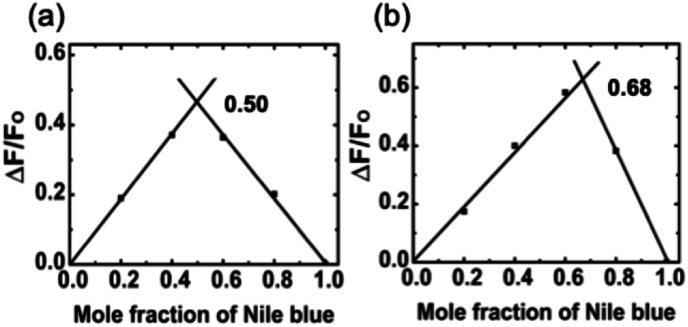
Job plot for Nile blue poly(A·U) complexation (a) and poly(I·C) (b) in 10 mM cacodylate buffer.

The binding stoichiometry and possible number of binding modes of Nile blue with the dsRNA were evaluated by continuous variation analysis (Job's plot). The Job's plot serves as a model-free approach for determining the stoichiometry of a specific complex. Multiple solutions are created, maintaining a constant total concentration of interacting partners while simultaneously varying the mole fraction ($X_{Nile\;blue}$). To assess the binding characteristics, we measured the fluorescence emission of Nile blue. The decrease in Nile blue fluorescence corresponded to complex formation, establishing a direct relationship between the fluorescence signal and complex formation. Sudden fluctuations in intensity signify the stoichiometry of the formed complex. While the simplicity of this method is appealing, caution must be exercised in ensuring the accuracy and identification of multiple complexes within the solution. The findings from the Job's plots depicted in Fig. 5 unequivocally reveal that a sole inflection point signifies a singular binding mode for Nile blue on the dsRNA polynucleotides. The determination of potential binding sites for Nile blue with poly(A·U) and poly(I·C) was subsequently calculated from the plot. Mole fraction for the intersection point for Nile blue-poly(A·U) was at 0.68, while for Nile blue-poly(I·C), it was at 0.50. The binding stoichiometry deduced from these data was 0.47 for poly(A·U) and 1.0 for poly(I·C), respectively. This is a novel finding that illustrates how base pair variations between RNA sequences influence binding dynamics.

### 3.4 Structural conformation analysis

**Fig. 6:**
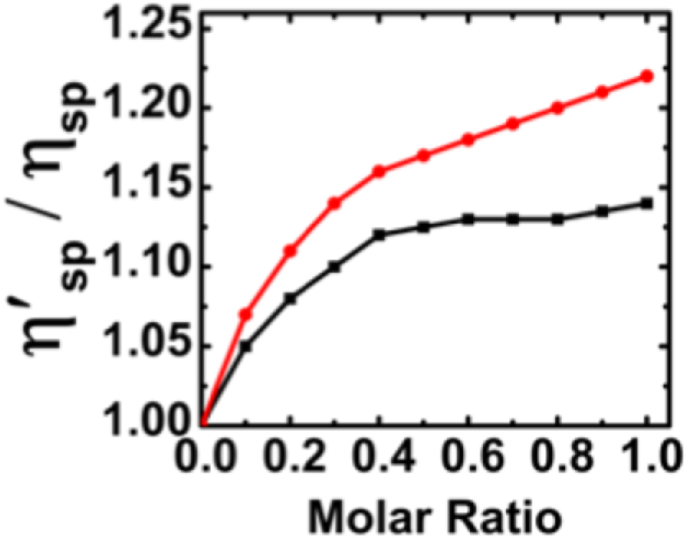
Relative specific viscosity of poly(A·U) (red) and poly(I·C) (black) with increasing concentration of Nile blue in 10 mM cacodylate buffer of pH 7.0 at 25 °C.

The hydrodynamic method was utilized to confirm the binding mode further. According to Lerman's assumption [45], it is postulated that the viscosity of the RNA solution increases upon complexation with an intercalator. If the dye molecules bind to double-stranded RNAs through intercalation or partial intercalation, the double-stranded RNAs not only alter the helical twist but also impact the contour length and stiffness. This increase in length becomes the primary factor contributing to changes in hydrodynamic parameters. Consequently, the relative specific viscosity of the RNA-dye compound rises with an increase in dye concentration, indicating the intercalation of dye molecules. This phenomenon occurs due to the enhancement of the axial length of the double helix, rendering it less flexible upon complexation and leading to an increase in the frictional coefficient. Therefore, viscosity serves as a hydrodynamic process employed to substantiate the intercalation binding mode. The viscosity of solutions containing poly(A·U) and poly(I·C) was assessed as Nile blue concentrations increased. As illustrated in the plot (Fig. 6), the relative viscosity of Nile blue-dsRNA complexes demonstrated an increase, indicating the stacking of Nile blue dyes between the RNA duplex and confirming the intercalation mode of binding. Notably, the viscosity changes were more pronounced when Nile blue was complexed with poly(A·U) compared to poly(I·C), aligning with the results obtained from fluorescence quenching studies.

**Fig. 7:**
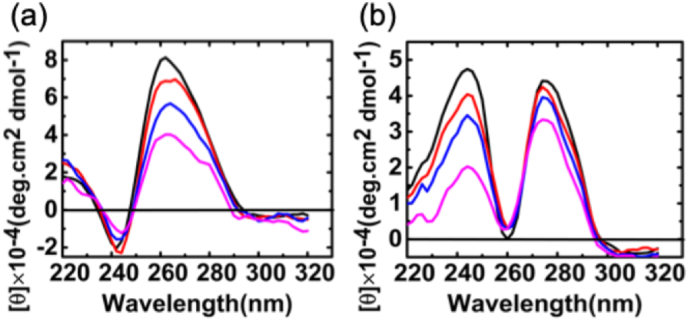
Circular Dichroism (CD) spectra of 10 μM poly(A·U) (a) and 10 μM poly(I·C) (b), both before (black) and after interacting with Nile blue at molar ratios of 0.5 (red), 1 (blue), and 1.5 (pink).

Circular dichroism (CD) spectroscopy serves as a crucial tool for identifying the secondary structure and structural alterations in nucleic acids. In this study, CD spectroscopy was employed to explore the conformational changes of RNA during the binding process. The CD spectra of poly(A·U) display a prominent positive peak in the 260–280 nm region, accompanied by a faint negative peak in the 240–260 nm region, in contrast, the CD spectra of poly(I·C) feature two positive peaks in the 220–260 nm and 260–300 nm regions [29]. The CD bands observed stem from the stacking interactions among the bases and helical conformations within the RNA duplex [46,47]. These interactions give rise to a chiral domain associated with the bases. While the specific base sequences may influence the exact characteristics and intensities, the overall patterns exhibit consistency [48]. Any discrepancies in CD spectra and ellipticity values are attributable to variations in the secondary structure of RNA resulting from interactions. The Nile blue-induced alterations in poly(A·U) and poly(I·C) conformation were recorded by testing the CD spectra within the 220–320 nm range while systematically varying the Nile blue/dsRNA mole ratio. The CD spectra obtained by adding Nile blue into solutions of poly(A·U) and poly(I·C) are displayed Fig. 7. The interaction between Nile blue and poly(A·U) led to a reduction in the CD ellipticity of the long-wavelength positive band, accompanied by a subtle increase in the CD ellipticity of the faint negative peak. The alterations in CD spectra of poly(I·C) associated with the interaction with Nile blue were marked by a reduction in intensity for both peaks. The changes in the CD ellipticity signify a modification in the number of base pairs per turn within the RNA helix, and the widening of the RNA groove through an increase in the winding angle. The CD spectroscopy results uncover significant conformational alterations in dsRNA upon binding, a new insight into how small molecules like Nile Blue modulate RNA secondary structures. This has potential implications for RNA-targeted therapeutic strategies.

### 3.5 Thermodynamic analysis

**Fig. 8:**
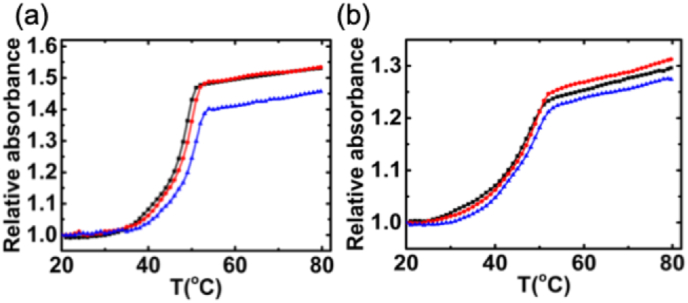
Thermal melting profiles of poly(A·U) (a) and poly(I·C) (b) in absence (black) of Nile blue and in presence of 10 μM Nile blue(red) and 15 μM Nile blue(blue).

Thermal denaturation profiles offer a convenient method for scrutinizing binding events and gauging the relative strengths of these interactions. The temperature at which the melting process reaches its midpoint is referred to as the melting temperature (Tm). At this temperature, fifty percent of the nucleic acid is in the helical form, while the remaining fifty percent exists in the single-stranded form. The extent to which a small molecule influences the nucleic acid's Tm relies on the nature of its interaction with the nucleic acid [49]. Typically, planar dye chromophores intercalating between RNA base pairs serve to stabilize the RNA helix, consequently elevating the Tm. The thermal melting profiles of poly(A·U) and poly(I·C) in the absence and presence of Nile blue are shown in Fig. 8. The T_m_ of the free poly(A·U) and poly(I·C) occurred at about 46.0 and 48.0 °C respectively. Upon interaction with Nile blue, the Tm of both poly(A·U) and poly(I·C) experienced an elevation; however, the degree of increase in poly(A·U) was more prominent compared to that in poly(I·C). The heightened degree of stabilization once again reinforces the notion of intercalation as the predominant mode of binding, with a stronger binding affinity observed for poly(A·U) compared to poly(I·C).

To gain a relevant insight into the type and magnitude of forces involved in the binding process, we investigated the change in constant pressure heat capacity (ΔC_P_) for each binding interaction. The ΔC_P_ values were determined by analyzing the slopes of the plots depicting the variation of ΔH with temperature (T):

$$\Delta C_{P}={\left(\frac{\partial \Delta H}{\partial T}\right)}_{p}$$

**Fig. 9:**
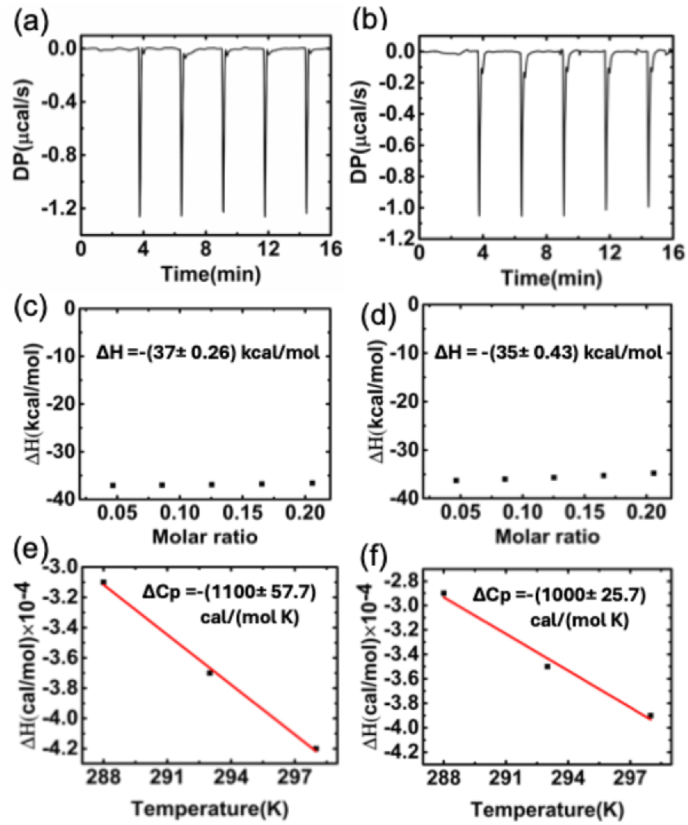
Heat burst patterns for excess-site titration of Nile blue to poly(A·U) (a) and poly(I·C) (b) at 293 K, along with the corresponding corrected integrated enthalpy (ΔH) determined for different molar ratios of Nile blue to poly(A·U) (c) and poly(I·C) (d) at 293 K. Heat capacity plot of Nile blue binding with to poly(A·U) (e) and poly(I·C) (f) obtained by performing ITC experiments at 288, 293, and 298 K.

The binding enthalpies (ΔH) were determined by excess-site method in isothermal titration calorimetry (ITC). This method involves utilizing a high concentration of nucleic acids to ensure that all introduced ligands bind to the host nucleic acid molecule in a stoichiometric manner. Meanwhile, at lower ligand concentrations, some undesired effects such as ligand self-association at higher concentrations can be minimized or eliminated to allow for the determination of “true” enthalpy values [50]. The integration of each peak should result in a consistent value for heat per injection. By adjusting the heat per injection signal relative to the total number of moles of ligand added per injection, independent estimations of binding enthalpy can be obtained without relying on binding models or biased fitting procedures. The resulting values of ΔH obtained from multiple titrations through this method constitute a significant sample size for averaging, thereby providing statistically reliable error estimates. The representative patterns of the heat burst curves obtained for each injection of Nile blue into poly(A·U) and poly(I·C) at 293 K are shown in Fig. 9a and b. The corrected enthalpy values (ΔH), obtained after subtracting the corresponding values from a blank experiment, for the interaction at different molar ratios of Nile blue to poly(A·U) and poly(I·C) at 293 K are presented in Fig. 9c and d, respectively. Additionally, data for three different temperatures have been deduced and are provided in Table 2.

**Table 2:** Binding enthalpies (ΔH) at different temperatures.

	ΔH/(kcal/mol) at 288K	ΔH/(kcal/mol) at 293K	ΔH/(kcal/mol) at 298K
poly(A·U)	- (31 ± 0.54)	-(37 ± 0.26)	-(42 ± 0.34)
poly(I·C)	-(29 ± 0.61)	-(35 ± 0.43)	-(39 ± 0.65)

Upon plotting ΔH against different temperatures, as shown in Fig. 9e and f, the heat capacity ΔC_P_ of Nile blue interacting with poly(A·U) and poly(I·C) were calculated as −1100 ± 57.7 cal/(mol.K) and −1000 ± 25.4 cal/(mol.K), respectively. Negative ΔC_P_ serves as a distinctive characteristic of small molecules binding to DNA and RNA. This phenomenon is linked to the release of structured water molecules, indicative of alterations in hydrophobic or polar group hydration. Negative ΔC_P_ is regarded as an indicator of the prevailing hydrophobic effect in the binding process. Furthermore, the ΔC_P_ values obtained in groove bonding model are expected to be lower compared to the typical values observed for DNA and RNA intercalators, therefore the magnitude of ΔC_P_ values also correlated to the structural change occurring during the binding process. Therefore, the more negative heat capacity values observed with the Nile blue-poly(A·U) interaction indicate a greater degree of interaction between Nile blue and poly(A·U) compared to poly(I·C), and the binding is driven by hydrophobic effect. The interaction is thermodynamically favored by negative enthalpy and is primarily driven by the hydrophobic effect. The demonstration of stronger interaction with poly(A·U) adds new dimensions to the biophysical understanding of RNA-small molecule interactions.

## 4 Conclusions

This study comprehensively investigated the binding of Nile blue to both poly(A·U) and poly(I·C) by intercalation, revealing distinct binding properties for different nucleic acid sequences. It was found that poly(A·U) exhibited a stronger affinity compared to poly(I·C). Obvious conformational changes were observed after intercalation binding. Thermodynamic analysis of the interaction revealed that binding was favored by negative enthalpy and negative ΔC_P_, indicating the involvement of significant hydrophobic forces in the complexation. These findings further advance our understanding of the interaction between the small molecule Nile blue and double-stranded RNA sequences, providing essential insights into potential therapeutic strategies and informing future material design.

## Declaration of competing interest

The authors declare that they have no known competing financial interests or personal relationships that could have appeared to influence the work reported in this paper.
